# Supplementary material for: Eye Movements in Silent Visual Speech Track Unheard Acoustic Signals and Relate to Hearing Experience
Source: eNeuro. 2025 Apr 25;12(4):ENEURO.0055-25.2025. doi: 10.1523/ENEURO.0055-25.2025 (PMC12037164; doi:10.1523/ENEURO.0055-25.2025)
Supplement: Table 1-1 — Group information. Download Table 1-1, DOCX file. [file eneuro-12-ENEURO.0055-25.2025-s001.docx]

|  | **Group** | Hearing | Congenitally deaf | Acquired DHH |
| --- | --- | --- | --- | --- |
|  | **Number of participants** | 49 | 7 | 19 |
| Sex | **Female** | 20 | 5 | 9 |
|  | **Male** | 29 | 2 | 10 |
| Age | **Mean** | 38 | 42 | 53 |
|  | **Standard deviation** | 14 | 13 | 9 |

*Supp. 1: Group information*
