# Supplementary figures and images for: Eye Movements in Silent Visual Speech Track Unheard Acoustic Signals and Relate to Hearing Experience
Source: eNeuro. 2025 Apr 25;12(4):ENEURO.0055-25.2025. doi: 10.1523/ENEURO.0055-25.2025 (PMC12037164; doi:10.1523/ENEURO.0055-25.2025)

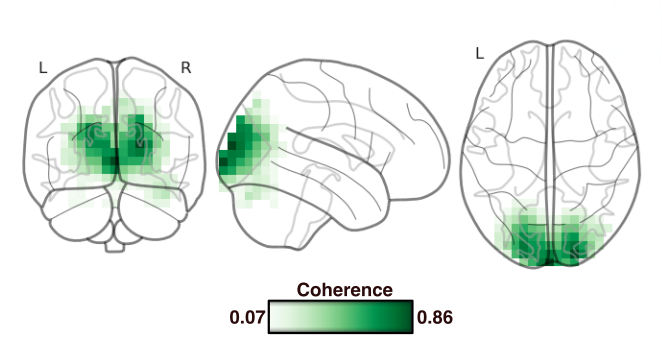

Supplement: Figure 1-1 — Sanity check for the source reconstruction: Voxel with the strongest lip coherence. Download Figure 1-1, TIF file. [file eneuro-12-ENEURO.0055-25.2025-s002.tif]

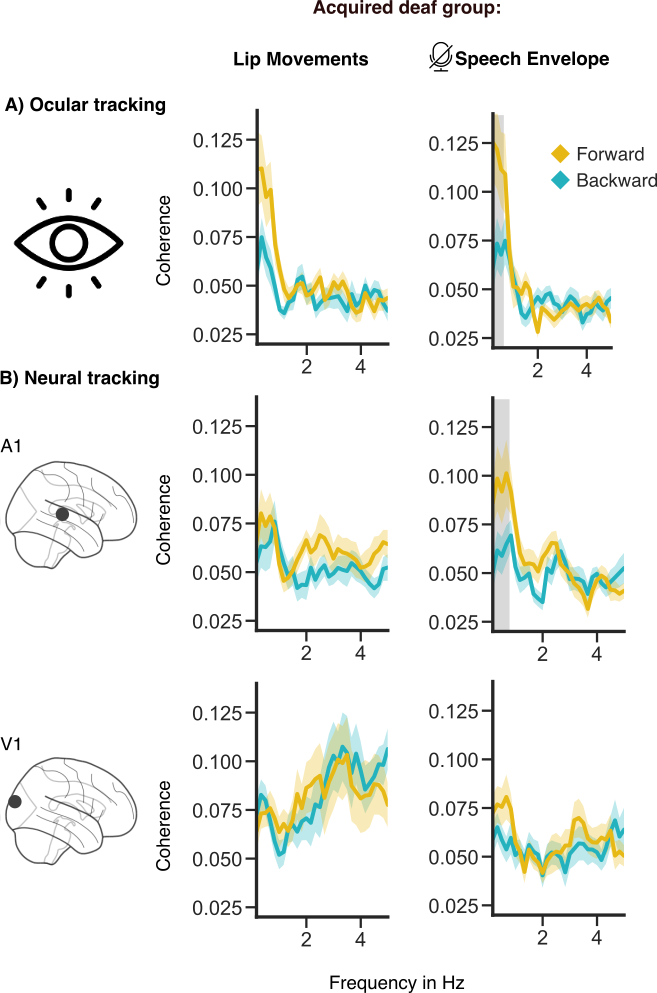

Supplement: Figure 1-2 — Effects for the acquired DHH group A) results of Coherence calculation of speech envelope and the lip movements with the selected ICA eye component. B) Coherence calculation at the strongest voxel in iii) in the primary auditory and visual cortex. Significant clusters are marked in gray (N = 19). Download Figure 1-2, TIF file. [file eneuro-12-ENEURO.0055-25.2025-s003.tif]

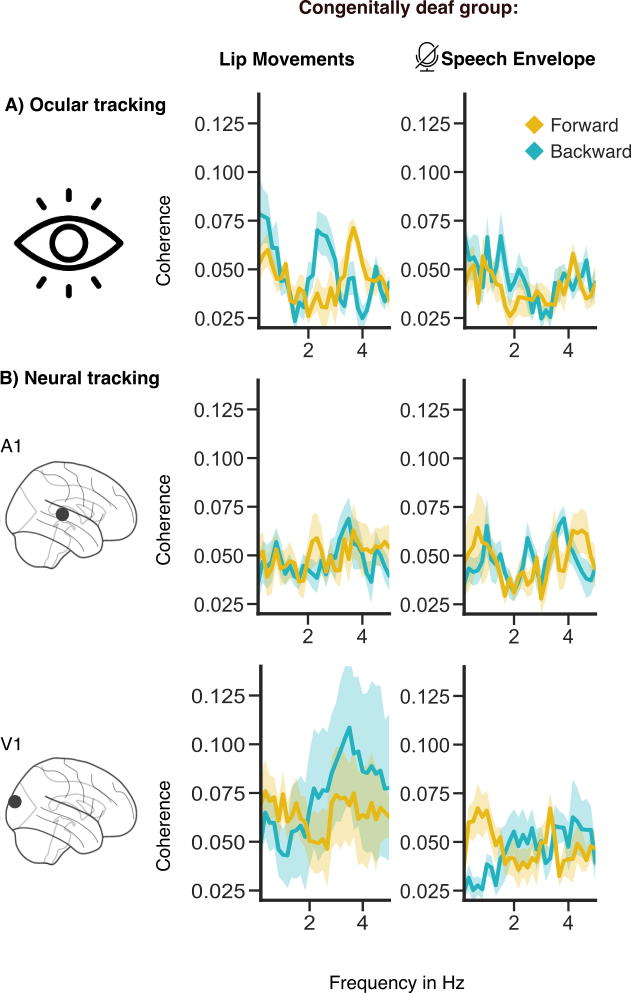

Supplement: Figure 1-3 — Effects for the acquired DHH group A) results of Coherence calculation of speech envelope and the lip movements with the selected ICA eye component. B) Coherence calculation at the strongest voxel in iii) in the primary auditory and visual cortex. Significant clusters are marked in gray (N = 7). Download Figure 1-3, TIF file. [file eneuro-12-ENEURO.0055-25.2025-s004.tif]

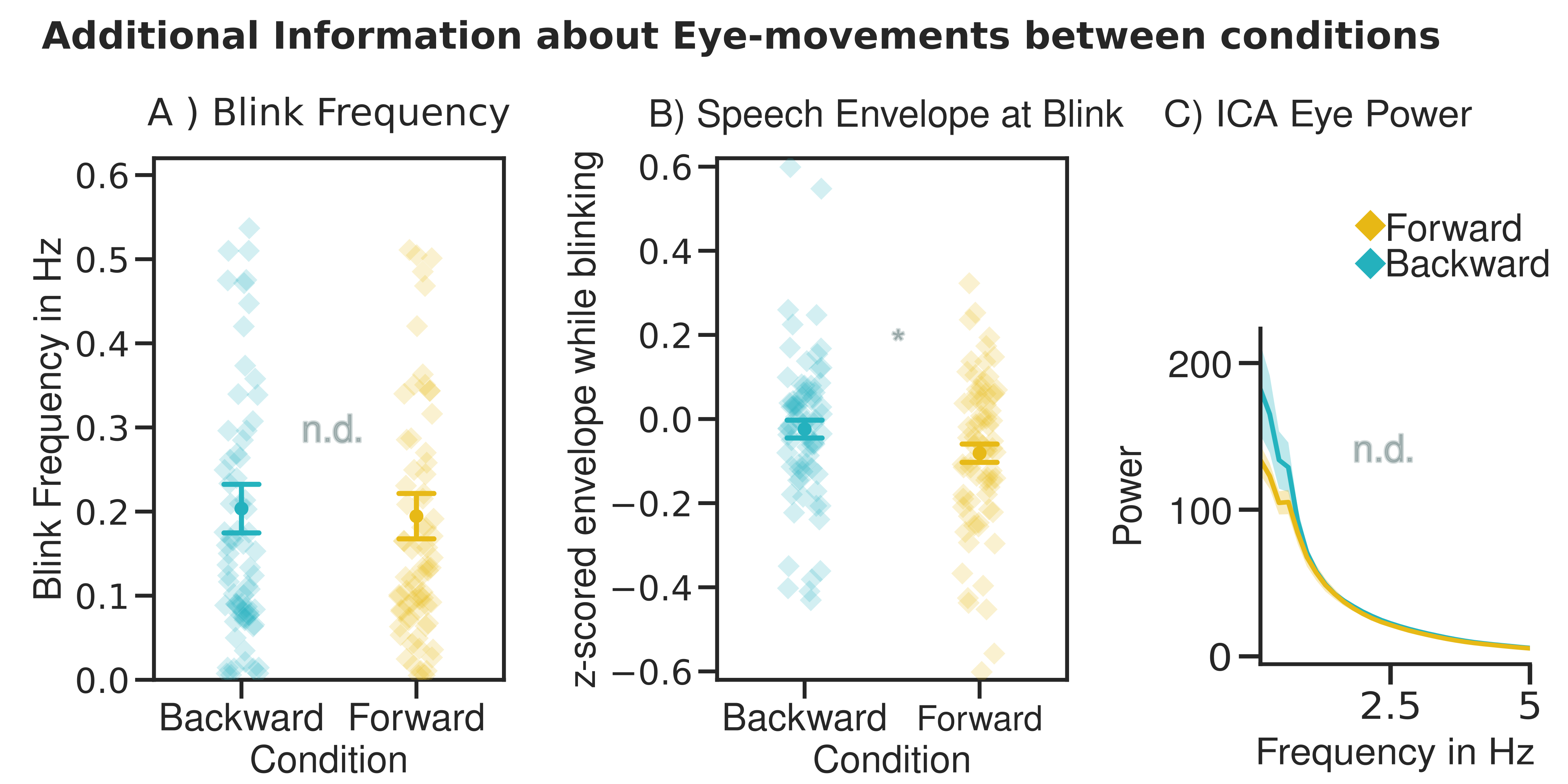

Supplement: Figure 1-4 — Additional information about the Eye-movements between conditions. A) The Blink frequency does not differ between the forward and the backward condition. (T(144) = -0.23, p = 0.82). The blinking frequency was 0.19 Hz in the Forward condition and 0.20 Hz in the Backward condition. B) The z-scored envelope value is lower in the forward condition compared to the backward condition (T(144) = -1.89, p < 0.05.) and overall, the envelope values are below zero (average) of the z-scored envelope (T(145)= -3.43, p < 0.001). C) The power of the ICA-Eye component does not differ significantly between the conditions, but the power in the backward condition is slightly increased in low frequencies. Download Figure 1-4, TIF file. [file eneuro-12-ENEURO.0055-25.2025-s006.tif]

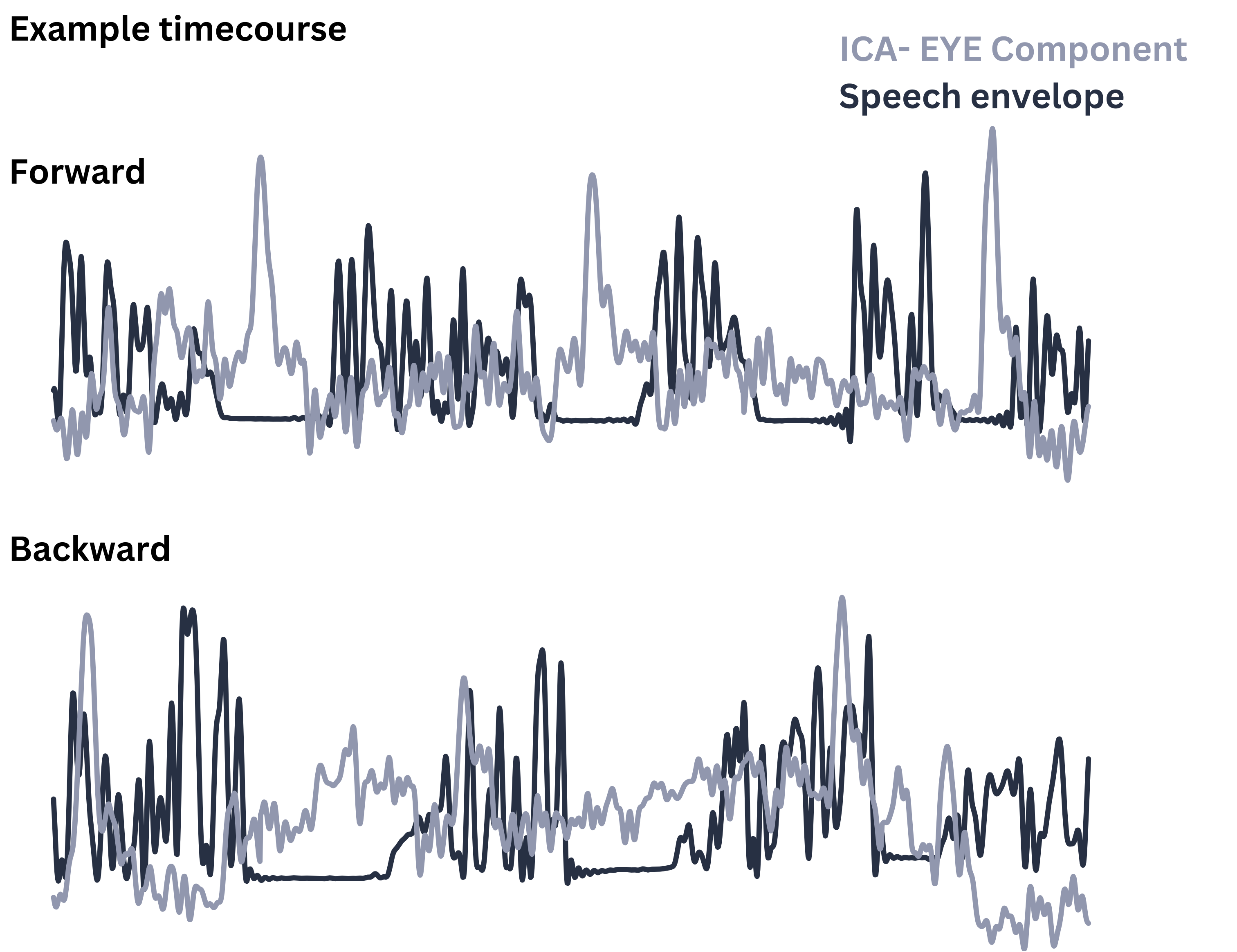

Supplement: Figure 1-5 — Exemplary 10 s time course of the participant with the highest ocular tracking. In the forward condition, the blinks appear to happen mostly, when the envelope is rather low. This is in line with Supp.6 B) showing lower speech envelope values while blinking in the forward condition compared to the backward condition. This participant has a blink frequency of 0.23 Hz in the forward condition and 0.25 Hz in the backward condition. Download Figure 1-5, TIF file. [file eneuro-12-ENEURO.0055-25.2025-s007.tif]

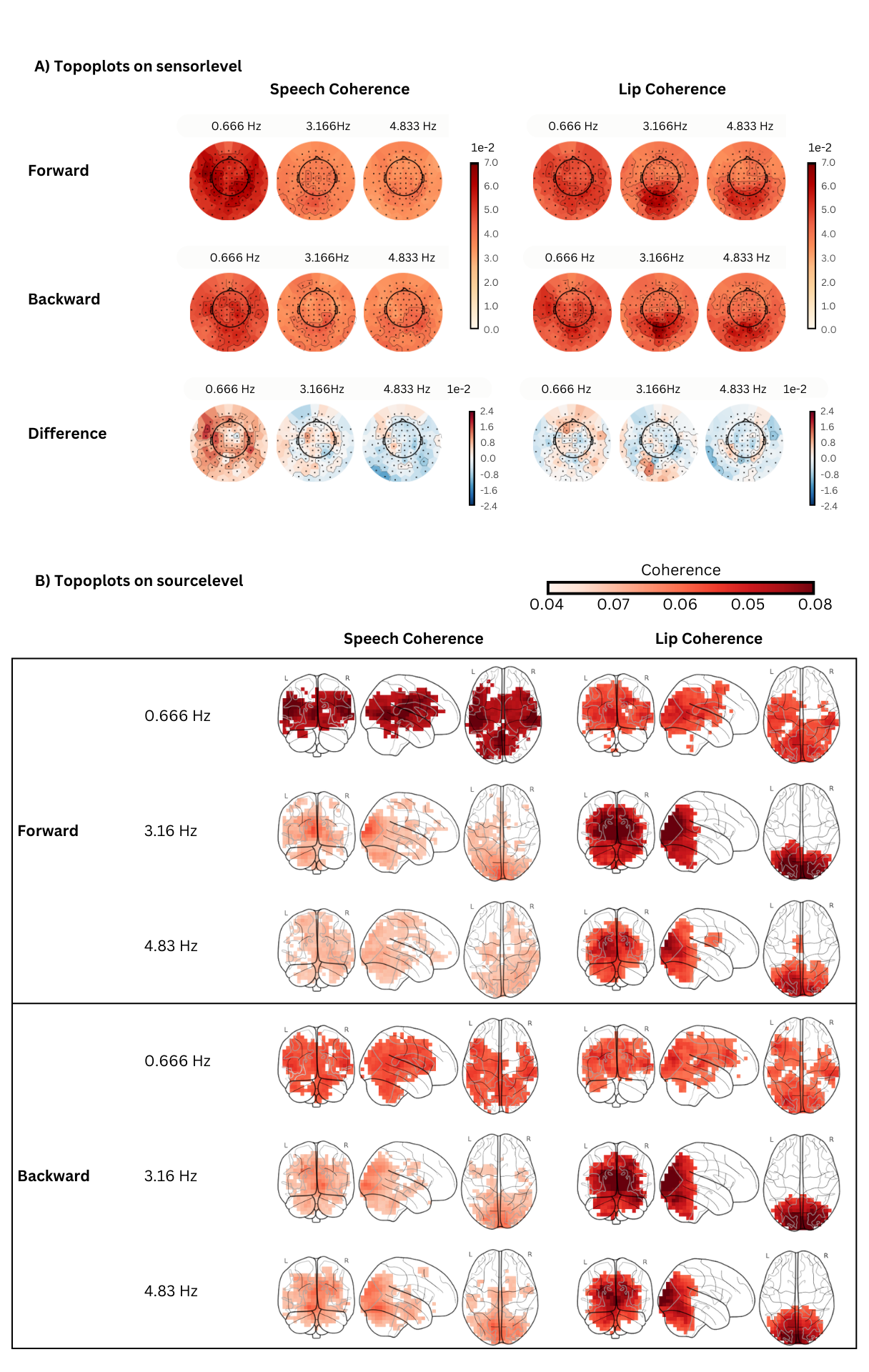

Supplement: Figure 2-1 — A) MEG sensor plot topographies of the Sensor- Speech and Sensor-Lip coherence in the forward condition, the backward condition. The difference is the backward condition subtracted from the forward condition. B) Forward solution of the topography in A). 20% of the highest voxels in coherence are presented. Download Figure 2-1, TIF file. [file eneuro-12-ENEURO.0055-25.2025-s005.tif]
